# Plasma-only circulating tumor DNA analysis detects minimal residual disease and predicts early relapse in hepatocellular carcinoma patients undergoing curative resection

**DOI:** 10.3389/fonc.2023.1119744

**Published:** 2023-03-07

**Authors:** Yuyan Xu, Jianpeng Cai, Kaihang Zhong, Yaohong Wen, Lei Cai, Guolin He, Hangyu Liao, Cheng Zhang, Shunjun Fu, Tingting Chen, Jinping Cai, Xuefeng Zhong, Chunzhu Chen, Mengli Huang, Yuan Cheng, Mingxin Pan

**Affiliations:** ^1^ Department of Hepatobiliary Surgery II, General Surgery Center, Zhujiang Hospital, Southern Medical University, Guangzhou, China; ^2^ Department of Pancreatobiliary Surgery, The First Affiliated Hospital, Sun Yat-Sen University, Guangzhou, China; ^3^ Medical Affairs, 3D Medicines, Inc., Shanghai, China

**Keywords:** hepatocellular carcinoma, circulating tumor DNA, minimal residual disease, plasma-only, early relapse

## Abstract

**Background:**

Minimal residual disease (MRD) is considered an essential factor leading to relapse within 2 years (early relapse) after radical surgery, which is challenging to be detected by conventional imaging. Circulating tumor DNA (ctDNA) provides a novel approach for detecting MRD and predicting clinical outcomes. Here, we tried to construct a fixed panel for plasma-only ctDNA NGS to enable tumor-uninformed MRD detection in hepatocellular carcinoma (HCC).

**Methods:**

Here, we performed the followings: (i) profiling genomic alteration spectrum of ctDNA from the Chinese HCC cohort consisting of 493 individuals by NGS; (ii) screening of MRD monitoring genes; and (iii) performance evaluation of MRD monitoring genes in predicting early relapse in the ZJZS2020 cohort comprising 20 HCC patients who underwent curative resection.

**Results:**

A total of 493 plasma samples from the Chinese HCC cohort were detected using a 381/733-gene NGS panel to characterize the mutational spectrum of ctDNA. Most patients (94.1%, 464/493) had at least one mutation in ctDNA. The variants fell most frequently in *TP53* (45.1%), *LRP1B* (20.2%), *TERT* (20.2%), *FAT1* (16.2%), and *CTNNB1* (13.4%). By customized filtering strategy, 13 MRD monitoring genes were identified, and any plasma sample with one or more MRD monitoring gene mutations was considered MRD-positive. In the ZJZS2020 cohort, MRD positivity presented a sensitivity of 75% (6/8) and a specificity of 100% (6/6) in identifying early postoperative relapse. The Kaplan-Meier analysis revealed a significantly short relapse-free survival (RFS; median RFS, 4.2 months *vs*. NR, P=0.002) in the MRD-positive patients versus those with MRD negativity. Cox regression analyses revealed MRD positivity as an independent predictor of poor RFS (HR 13.00, 95% CI 2.60-69.00, P=0.002).

**Conclusions:**

We successfully developed a 13-gene panel for plasma-only MRD detection, which was effective and convenient for predicting the risk of early postoperative relapse in HCC.

## Introduction

Liver cancer is the second leading cause of cancer death, and its incidence is growing worldwide (1–4). Over a million people per year are expected to be liver cancer patients by 2025 (5). Hepatocellular carcinoma (HCC) is the main type of primary liver cancer, comprising approximately 90% of patients. Although hepatectomy is a widely accepted treatment option for HCC patients with good liver function, the relapse rate of up to 60%-70% within 5 years after surgery remains a severe problem (6–8).

Relapse in multiple solid tumors, including HCC, can be divided into early relapse (≤2 years) mainly caused by minimal residual disease (MRD) following resection, and late relapse (>2 years) caused by *de novo* tumors arising in a microenvironment predisposed to carcinogenesis (9–14). Early relapse has been reported to account for over 60% of all relapsed HCC events (9, 15–19). Consequently, there has been much interest in detecting and eliminating MRD to prevent relapse or for early treatment of degeneration. Current postoperative surveillance methods are not sensitive or specific enough to detect MRD, such as monitoring clinical symptoms, tumor markers, and routine imaging. As a result, improving current therapeutic strategies and preventing recurrence might be achieved by establishing more precise MRD detection approaches.

Circulating cell-free DNA (cfDNA) is extracellular nucleic acid fragments released into the bloodstream due to apoptosis and necrosis from both healthy and malignant cells. There has been strong evidence that postoperative tumor-derived cfDNA (ctDNA) detection is correlated with MRD and could identify patients at high risk of relapse (20–23). Currently, there are two available ctDNA detection strategies for monitoring MRD: tumor-informed and tumor-uninformed (also referred to as tumor-agnostic, tumor-naïve, or plasma-only) assay.

The tumor-informed approach relies on tumor tissue sequencing to identify tumor-derived alterations for the design of patient-specific targeted gene panels for ctDNA tracking, which has presented effectiveness for monitoring MRD in some solid tumors after curative-intent treatment, including colon, lung, and pancreas cancer (24–27). A potential limitation is that tumor heterogeneity in both space and time can affect its performance and might generate some false negative results (28, 29). Additionally, designing individualized next-generation sequencing (NGS) panels also lengthens the turnaround time versus the fixed NGS panel. The tumor-agnostic approach only requires plasma cfDNA sequencing with a fixed panel, which endows it with the advantages of noninvasiveness, convenience, cost-effectiveness, and rapid turnaround time. The method’s drawback is the lack of sensitivity, which can be improved by incorporating serial longitudinal surveillance samples and examining a variety of biomarkers, such as ctDNA mutation and ctDNA methylation. Given the attractive advantages and redeemable disadvantages, this approach has increasingly been investigated in multiple solid tumors. Excitingly, one recent study reported that plasma-only MRD detection presented favorable sensitivity and specificity for predicting recurrence in colorectal cancer patients undergoing curative-intent surgery, comparable to the tumor-informed approach (30). However, few studies have examined whether a plasma-only ctDNA assay can identify MRD in HCC with clinically meaningful specificity and sensitivity.

In this study, we first performed a plasma-only ctDNA assay integrating genomic signatures to identify gene candidates associated with MRD in two cohorts. We then determined whether these genes could reliably identify MRD and predict early relapse in postoperative patients with HCC.

## Materials and methods

### Study design and population

There are three major phases in this study: (i) profiling genomic alteration spectrum of HCC using ctDNA from the Chinese HCC cohort; (ii) screening of MRD monitoring genes *via* excluding the genes mutated at high frequency in ctDNA of the ZJ2020 cohort with patients having relapse-free survival (RFS) more than 2 years after radical resection from the comprehensive mutational spectrum of ctDNA in the Chinese HCC patient cohort; and (iii) performance evaluation of MRD monitoring genes in predicting early relapse in the ZJZS2020 cohort comprising HCC patients underwent curative surgical resection. The study design and the CONSORT participant flow diagram are summarized in **Supplementary Figure 1**.

The Chinese HCC patient cohort consisted of 493 patients from the Zhujiang Hospital of Southern Medical University and the First Affiliated Hospital, Sun Yat-Sen University, who underwent plasma-based NGS using a 381/733-gene panel (3D Medicines Inc. Shanghai, China) between January 6, 2017 and June 2, 2020. The gene lists of these two panels were attached in **Supplementary Tables 1**, **2**. The Venn diagram revealed 294 shared genes between these two panels (**Supplementary Figure 2**).

The ZJ2020 cohort included 24 operable HCC patients who underwent radical hepatectomy at the Zhujiang Hospital of Southern Medical University from May 25, 2010 to October 23, 2020, and remained disease-free for greater than two years postoperatively. Of 24 patients, 10 received transcatheter arterial chemoembolization (TACE) after surgery. The blood samples (≥10 ml per case) were collected within 1-4 weeks after surgery or completion of TACE if they received TACE. The plasma ctDNA was analyzed using a 381/733-gene NGS panel.

The ZJZS2020 cohort consisted of 20 operable HCC patients who had curative resection at the Zhujiang Hospital of Southern Medical University from July 8, 2019 to December 28, 2020. The blood samples were collected about one week after surgery and submitted to NGS with a 381/733-gene panel.

In the two surgery cohorts, ZJ2020 and ZJZS2020, clinicopathological data were collected through review of the medical records using a standardized case report form, including age, sex, liver function, tumor stage, maximum tumor diameter, tumor number, etiology, alpha-foetoprotein (AFP) level, microvascular invasion (MVI), and portal vein tumor thrombus (PVTT).

This study was conducted following the principles of the Declaration of Helsinki and approved by the Ethics Committee of the Zhujiang Hospital of Southern Medical University (Approval number 2022-KY-150-01). Written informed consent was obtained from all participants in this study.

### Blood sample processing and cfDNA isolation

Whole blood (2 × 10 ml) from every patient was drawn into cell-free DNA BCT tubes (Streck). Blood samples were centrifuged, and cfDNA was extracted and quantified using the QiAmp Circulating Nucleic Acid Kit (Qiagen) and the Qubit dsDNA HS Assay Kit (Thermo Fisher Scientific), respectively, as per the manufacturer’s instructions.

### Library preparation and targeted capture

cfDNA libraries were constructed and barcoded with unique molecular identifiers (UMI). The libraries were then PCR-amplified and purified for target enrichment. For targeted capture, indexed libraries were subjected to probe-based hybridization with the customized NGS panel.

### DNA sequencing, data processing, and variant calling

The captured libraries were subjected to 100-bp paired-end sequencing on an Illumina NovaSeq 6000. The average effective sequencing depth for the 381-gene and 733-gene NGS panels was 5161× (range, 1840-10664) and 6337× (range, 2194-14250), respectively. Raw reads were mapped to the reference human genome hg19. An in-house developed software was applied to generate a duplex consensus sequence by incorporating a dual UMI at the end of the DNA fragments. An in-house loci-specific variant detection model based on a binary test was also used to improve specificity, especially for variants with low allele frequency. Following these steps, the variants were filtered by supporting read count, strand bias, base quality, and mapping quality. Variant calling was also optimized to identify variants at short tandem repeat regions. Single-nucleotide polymorphism (SNPs) and indels were annotated using ANNOVAR. Only missense, stopgain, frameshift, and non-frameshift indel mutations were retained.

### Statistical analysis

Gene mutation analysis was performed by R package maftools (31). Wilcoxon test was adopted to estimate differences between continuous variables. The difference in proportions between groups was assessed using the Chi-square test. Kaplan-Meier analysis with a log-rank test was conducted to compare RFS between groups. Cox regression analyses were applied to identify variables associated with RFS. Statistical analysis was performed using R version 4.0.3. All results were considered statistically significant when the P value was less than 0.05.

## Results

### Mutational landscape in HCC by ctDNA profiling

**Figure 1:**
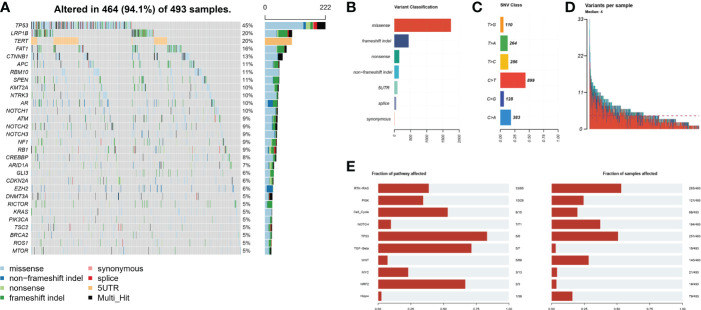
Mutational landscape by ctDNA profiling in the Chinese HCC cohort. **(A)** The ratio of the top 30 genes in the number of mutations. **(B)** Frequency of different mutation classifications. **(C)** Frequency of SNV class. **(D)** Variant number per HCC samples. **(E)** The number of mutated genes contained in the number of mutated samples per pathway. HCC, hepatocellular carcinoma; SNV, single nucleotide variation.

**Figure 2:**
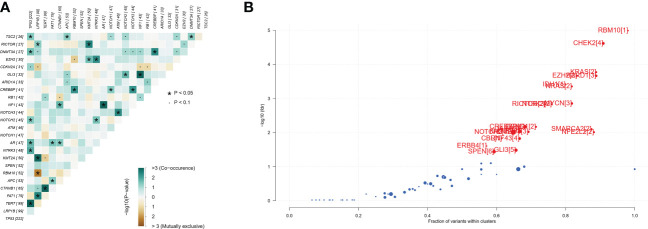
Identification of mutation interactions and cancer driver genes in the Chinese HCC cohort. **(A)** The co-occurrence or exclusive associations between top 25 mutation genes. **(B)** Detecting cancer driver genes based on positional clustering in HCC. Each dot represents a gene, and the size of the dot represents the number of clusters (mentioned inside square brackets) within which a fraction (X-axis) of total variants is accumulated. HCC, hepatocellular carcinoma.

A total of 493 plasma samples from Chinese patients with HCC were detected to characterize the mutational spectrum of ctDNA, wherein 285 plasma samples were tested with the 381-gene panel, and 208 were determined with the 733-gene panel. Most patients (94.1%, 464/493) had at least one mutation in ctDNA. The top 30 mutated genes are shown in **Figure 1**. The top five most frequently mutated genes were *TP53* (45.1%), *LRP1B* (20.2%), *TERT* (20.2%), *FAT1* (16.2%), and *CTNNB1* (13.4%) (**Figure 1A**). Variant classification showed that the missense mutations were the most common (**Figure 1B**). SNPs were the most common variant types. Among the single-nucleotide variation (SNV) class, C > T was the most common base substitution (**Figure 1C**). The median variant number per sample was 4 (range, 1-33) (**Figure 1D**). The pathway analysis showed that the top affected pathways were TP53, TGF-β, NRF2, Cell_cycle, RTK-RAS, and PI3K which occurred in a substantial number of HCC plasma samples (**Figure 1E**). Besides, the analysis of mutation interactions identified one mutually exclusive and 28 co-occurring events in the top 25 highly mutated genes (**Figure 2A**). We also used the OncodriveCLUST algorithm to identify potential driver mutations in HCC. The top six driver genes ordered according to z-score were *RBM10*, *CHEK2*, *KRAS*, *BARD1*, *EZH2*, and *IDH1* (**Figure 2B**).

### Screening of MRD monitoring genes

**Figure 3:**
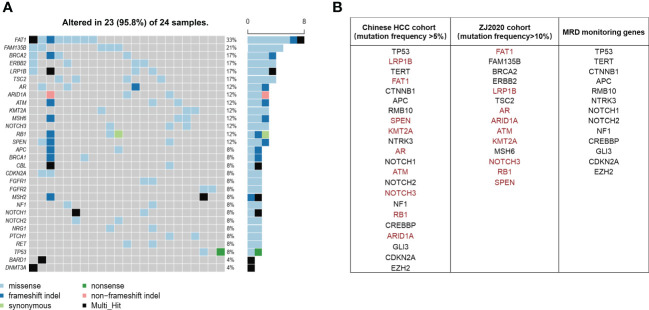
Screening of MRD monitoring genes. **(A)** The top 30 frequently mutated genes in the ZJ2020 cohort with HCC patients having RFS more than 2 years after radical resection. **(B)** The 14 genes with a mutation frequency of >10% in the ZJ2020 cohort are filtered out from the 27 genes with a mutation frequency of >5% in the Chinese HCC cohort, and the remaining 13 genes are used as MRD monitoring genes. HCC, hepatocellular carcinoma; MRD, minimal residual disease; RFS, relapse-free survival.

It has been well documented that early relapse after surgery is primarily due to MRD (9–12). Thus we subtracted the genes mutated at high frequency in ctDNA of the ZJ2020 cohort from the comprehensive mutational spectrum of ctDNA in the Chinese HCC cohort to screen for MRD monitoring genes. Baseline characteristics of the ZJ2020 cohort are listed in **Supplementary Table 3**. This cohort comprised 24 HCC patients with RFS more than 2 years after radical resection. As of May 8, 2019, the median postoperative follow-up was 35.5 months. Twenty-one patients remained on RFS, and three experienced recurrence at postoperative 34.7, 46.4, and 52.8 months, respectively. We identified ≥1 mutation in 23 of 24 (95.8%) patients. The most frequently mutated genes included *FAT1*, *FAM135B*, *BRCA2*, *ERBB2*, *LRP1B*, *TSC2*, *AR*, *ARID1A*, *ATM*, *MSH6*, *NOTCH3*, *RB1*, and *SPEN*, with each being mutated in at least 10% of patients (**Figure 3A**). These 14 genes were considered to be unrelated to early relapse and were filtered out from the 27 genes with a mutation frequency of > 5% in the Chinese HCC cohort (32). The remaining 13 genes were regarded as MRD monitoring genes. As shown in **Figure 3B**, these 13 genes were *TP53*, *TERT*, *CTNNB1*, *APC*, *RMB10*, *NTRK3*, *NOTCH1*, *NOTCH2*, *NF1*, *CREBBP*, *GLI3*, *CDKN2A*, and *EZH2*. Any plasma sample with one or more MRD monitoring gene mutations was considered MRD-positive and otherwise MRD-negative (33, 34).

### The predictive value of MRD status for early relapse

**Figure 4:**
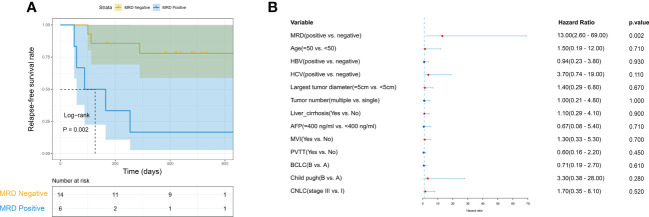
The predictive value of MRD status for early relapse. **(A)** Kaplan-Meier curves comparing RFS between MRD-positive and -negative patients with HCC. **(B)** The univariate Cox regression analyses reveal that only MRD status is significantly associated with RFS. HCC, hepatocellular carcinoma; MRD, minimal residual disease; RFS, relapse-free survival.

We further evaluated the predictive value of MRD status for early postoperative relapse in the ZJZS2020 cohort comprising 20 HCC patients who underwent R0 resection. **Table 1** presents the baseline characteristics of this cohort. Patients were predominantly male (n=17, 85%), and the median age was 57.5 years (range, 42-76). Most patients presented HBV infection (14/20, 70.0%), Child-Pugh A (19/20, 95.0%), and a single tumor (15/20, 75.0%). About half of the patients had cirrhosis (11, 55.0%) and MVI (12/20, 60.0%) and were at BCLC stage C (10/20, 50.0%). The AFP level of ≥ 400 ng/mL was observed in three patients (15.0%). We identified ≥ 1 mutation in 19 of 20 (95.0%) patients. Based on the definition of MRD status, 20 patients were divided into two subgroups, MRD-positive (6, 30%) and MRD-negative (14, 70%) subgroups. The specific mutational information of 6 MRD-positive patients is listed in **Table 2**. Patient baseline characteristics were well balanced between subgroups. With a median postoperative follow-up of 14.1 months (range, 5.5-22.1 months), six MRD-positive cases and two of 14 MRD-negative cases had experienced an early relapse. MRD status presented a sensitivity of 75% (6/8) and a specificity of 100% (6/6) in identifying early postoperative relapse. Kaplan-Meier plots indicated that the MRD-positive patients had a significantly poor RFS (mRFS, 4.2 *vs*. NR months, P=0.002) compared with the MRD-negative patients (**Figure 4A**). Univariable analysis revealed that MRD status was the only significant variable to predict PFS (HR=13.00, 95% CI 2.60-69.00, P=0.002) (**Figure 4B**).

**Table 1:** Basic characteristics of HCC patients in the ZJZS2020 cohort.

Clinicopathologic factors	MRD-negative (N=14)	MRD-positive (N=6)	Total (N=20)	P value
**Sex**				0.231
Female	3 (21.4%)	0 (0.0%)	3 (15.0%)	
Male	11 (78.6%)	6 (100.0%)	17 (85.0%)	
**Age**				0.231
<50	3 (21.4%)	0 (0.0%)	3 (15.0%)	
≥50	11 (78.6%)	6 (100.0%)	17 (85.0%)	
**HBV**				0.213
Negative	3 (21.4%)	3 (50.0%)	6 (30.0%)	
Positive	11 (78.6%)	3 (50.0%)	14 (70.0%)	
**HCV**				0.026
Negative	14 (100.0%)	4 (66.7%)	18 (90.0%)	
Positive	0 (0.0%)	2 (33.3%)	2 (10.0%)	
**Largest tumor diameter**				0.583
<5cm	4 (28.6%)	1 (16.7%)	5 (25.0%)	
≥5cm	10 (71.4%)	5 (83.3%)	15 (75.0%)	
**Tumor number**				0.583
Multiple	3 (21.4%)	2 (33.3%)	5 (25.0%)	
Single	11 (78.6%)	4 (66.7%)	15 (75.0%)	
**Liver cirrhosis**				0.774
No	6 (42.9%)	3 (50.0%)	9 (45.0%)	
Yes	8 (57.1%)	3 (50.0%)	11 (55.0%)	
**AFP**				0.231
≤400 ng/ml	11 (78.6%)	6 (100.0%)	17 (85.0%)	
>400 ng/ml	3 (21.4%)	0 (0.0%)	3 (15.0%)	
**MVI**				0.56
No	5 (35.7%)	3 (50.0%)	8 (40.0%)	
Yes	9 (64.3%)	3 (50.0%)	12 (60.0%)	
**PVTT**				0.214
No	5 (35.7%)	4 (66.7%)	9 (45.0%)	
Yes	9 (64.3%)	2 (33.3%)	11 (55.0%)	
**BCLC**				0.239
A	3 (21.4%)	0 (0.0%)	3 (15.0%)	
B	5 (35.7%)	2 (33.3%)	7 (35.0%)	
C	6 (42.9%)	4 (66.7%)	10 (50.0%)	
**Child pugh**				0.127
A	14 (100.0%)	5 (83.3%)	19 (95.0%)	
B	0 (0.0%)	1 (16.7%)	1 (5.0%)	
**CNLC**				0.836
I	4 (28.6%)	2 (33.3%)	6 (30.0%)	
III	10 (71.4%)	4 (66.7%)	14 (70.0%)	

HBV, hepatitis B virus; HCV, hepatitis C virus; AFP, alpha-foetoprotein; MVI, microvascular invasion; PVTT, portal vein tumor thrombosis; BCLC, Barcelona Clinic Liver Cancer Staging; CNLC, China Liver Cancer Staging.

**Table 2:** The specific mutational information of six MRD-positive HCC patients.

ID	Gene	Chromosome	Exon	c.dot	p.dot	frequency	Variant Type
69168	CREBBP	chr16	Exon31+1424	c.6596A>T	p.Q2199L	0.003217	Substitution
69168	EZH2	chr7	Exon10-30	c.1209_1211del	p.E404del	0.00552	Deletion
69168	TP53	chr17	Exon5+1	c.376T>G	p.Y126D	0.003066	Substitution
79060	TP53	chr17	Exon6+48	c.607G>A	p.V203M	0.002152	Substitution
52813	NF1	chr17	Exon47-68	c.6995C>G	p.S2332*	0.005093	Substitution
63144	EZH2	chr7	Exon2-56	c.62C>G	p.S21*	0.003297	Substitution
63144	GLI3	chr7	Exon15+1996	c.4427del	p.N1476Tfs*12	0.003307	Deletion
63144	NF1	chr17	Exon3-13	c.276del	p.K92Nfs*11	0.003922	Deletion
65536	APC	chr5	Exon16-3556	c.7090del	p.M2364Cfs*10	0.003487	Deletion
65536	GLI3	chr7	Exon15+1996	c.4427del	p.N1476Tfs*12	0.003692	Deletion
92916	TERT	chr5		c.-124C>T		0.010394	Substitution
92916	TP53	chr17	Exon7-36	c.747G>T	p.R249S	0.010289	Substitution
92916	TP53	chr17	IVS3-2	c.97-2A>T		0.00618	Substitution

HCC, hepatocellular carcinoma; MRD, minimal residual disease.

### ctDNA dynamic monitoring

**Figure 5:**
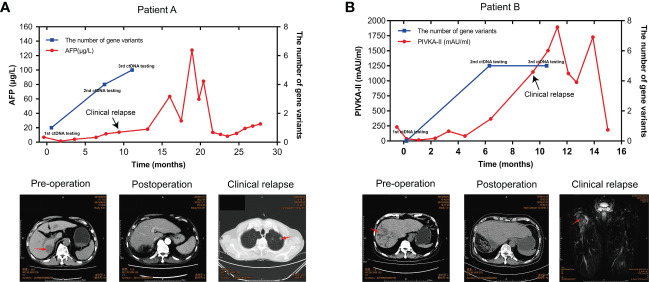
ctDNA dynamic monitoring. The dynamical changes in the number of MRD monitoring gene variants, the level of AFP/PIVKA-II in plasma samples, and imaging results of HCC patients A **(A)** and B **(B)** during HCC progression. HCC, hepatocellular carcinoma; MRD, minimal residual disease; AFP, alpha-foetoprotein; PIVKA-II, a protein induced by vitamin K absence or antagonist II.

Of 20 patients in the ZJZS2020 cohort, two patients (Patients A and B) underwent longitudinal assessment of MRD status (**Figures 5A, B**). Patient A is a 76-year-old male with HCV positivity, and his surgical pathological examination demonstrated a grade II-III HCC with MVI and no definite nerve invasion. AFP was restored to the normal level from 7.05ug/L before the operation. The blood collected seven days after surgery was subjected to ctDNA analysis using the 381-gene panel. *NF1* mutation with a variant allele fraction (VAF) of 0.0050933 was observed in the plasma sample, suggesting that this patient was MRD-positive. This patient was subsequently followed up, and his AFP level was examined every 1-2 months. On postoperative month 8, the AFP level increased to 11.68 ug/L; ctDNA analysis presented the mutations in three MRD monitoring genes (*NF1*, *TP53*, and *CTNNB1*) with the maximal VAF of 0.0345581; and the imaging evaluation showed no sign of abnormality. On postoperative month 9.6, computed tomography (CT) scanning revealed multiple metastases of both lungs and mediastinal lymph node metastases, and AFP level was increased up to13.97 μg/L, suggesting disease progression. Meanwhile, ctDNA analysis identified mutations in four monitoring genes, including *TERT*, *TP53*, *CTNNB1*, and *EZH2*, and the maximal VAF of 0.232117 was obviously higher than that of the previous two tests.

Patient B is a 57-year-old male with HBV-positive liver cirrhosis. The postoperative pathology revealed a grade II-III HCC with MVI and hepatic capsule invasion. The analysis of ctDNA from the plasma collected seven days after surgery showed no mutations in the MRD monitoring genes, and the patient was MRD-negative. *CREBBP* mutation with a VAF of 0.0050933 was detected by ctDNA analysis on postoperative month 6, suggesting that the patient’s MRD status converted from negative to positive, while the imaging evaluation showed no sign of abnormality. The AFP level remained normal after surgery. The PIVKA-11, a potential biomarker complementary to AFP for HCC diagnosis, remained stable in the first six months after surgery and then increased rapidly, reaching 1146.71 mAU/ml in the tenth postoperative month. At 11 months after surgery, the PET-CT imaging results showed changes in the upper part of the right femur and increased metabolism, which was more considered for metastasis and disease recurrence; the ctDNA results still represented MRD-positive. These results showed that serial ctDNA monitoring provides dynamic information on somatic variants with pathogenic effects in tumors and might be superior to single detection to evaluate disease progression.

## Discussion

In this study, we profiled genomic alterations by targeted NGS of ctDNA from Chinese HCC patients and identified MRD monitoring genes by filtering out the genes unrelated to early relapse to define MRD status. Evaluation of the prognostic value of MRD status in an independent cohort showed that MRD positivity was an independent prognostic factor for poor RFS. Additionally, the data of two patients undergoing multiple ctDNA tests suggest that the serial longitudinal MRD status tracking may be superior to single testing in predicting postoperative relapse of HCC patients.

Genomic profiles of ctDNA have been shown to be closely associated with types and stages of malignant tumors, suggesting that ctDNA could contribute to cancer diagnosis and personalized treatment (35, 36). The genomic characterization of plasma ctDNA of HCC patients has been reported by several studies, but these studies focus primarily on limited genes or in a small subset. For instance, in Caucasian populations, IKEDA et al. (37) reported the spectrum of genomic mutations in ctDNA from 14 HCC patients by a 68-gene NGS panel, and all patients had somatic alterations, of which the most predominant somatic mutations were in *TP53* (57%), followed by *CTNNB1* (29%), *PTEN* (7%), *CDKN2A* (7%), *ARID1A* (7%), and *MET* (7%). In Chinese populations, Cai and colleagues (38) analyzed the mutation profiles of plasma ctDNA from three HCC patients by a 574-gene NGS panel, highlighting that ctDNA may be able to overcome tumor heterogeneity and monitor therapeutic efficacy in real time. Yan et al. (39) utilized a 354-gene NGS panel to profile the genomic landscape of ctDNA from 26 HCC patients and found a positive ctDNA detection rate of 96.2%, wherein *TP53* (50.00%) was the most common mutant gene, followed by *AXIN1* (11.54%), *BCOR* (11.54%), *CTNNB1* (11.54%), *FANCE* (11.54%), *FANCM* (11.54%), and *NCOR1* (11.54%). Here we characterized the genomic alteration spectrum of ctDNA from 493 Chinese HCC patients by the 381/733-gene NGS panel. To our knowledge, this is the most comprehensive targeted NGS analysis of ctDNA in Chinese HCC. Consistently, *TP53* was the most commonly mutated gene (45%). It is worth noting that the detection rate of ctDNA in the above studies of others and ours is higher than 90%. The high ctDNA detection rate may be attributed to the high vascular nature of HCC or/and the decreased hepatic clearance of ctDNA, because the liver is the main organ responsible for cfDNA clearance (40). Meanwhile, the high ctDNA detection rate also suggested that NGS-based ctDNA detection is technically feasible for identifying genomic variations in patients with HCC. Furthermore, the mutation profiling of ctDNA showed similar mutation frequencies for multiple known HCC drivers, including *TP53*, *TERT*, *CTNNB1*, *CDKN2A*, *ARID1A*, *RB1*, and *LRP1B* (41–43), compared to those of tissue samples reported by Wang et al. (44), supporting biological plausibility and feasibility of plasma ctDNA testing among patients with HCC.

MRD is considered an important factor generating early postoperative relapse, and ctDNA detection based on the tumor-informed or tumor-agnostic approach has been proven an effective method for detecting MRD in various tumor types (24–27, 30, 45, 46). While the published research on ctDNA detection in HCC is mainly based on tumor-informed approaches. For example, Zhu et al. (20) applied a tumor-informed ctDNA detection approach in HCC patients by a 197-gene panel and identified that postoperative ctDNA positivity was significantly associated with tumor recurrence. Similarly, Cai et al. (14) identified that postoperative ctDNA could indicate MRD more accurately than the conventional protein biomarkers AFP and DCP and was an independent prognostic factor for both RFS and overall survival. These findings show that tumor-informed ctDNA testing is feasible for monitoring MRD, but its broad application is inevitably constrained by the inherent limitations of the strategy, such as the inability to detect emerging mutations, long and much labor-intensive processing, and the resulting high cost. Here we tried to construct a fixed panel to detect MRD. The MRD monitoring genes that made up this fixed panel were identified by removing the genes with a mutation frequency of ≥ 10% in ctDNA of the ZJ2020 cohort with patients having RFS over 2 years after radical resection from the genes with a mutation frequency of ≥ 5% in ctDNA of Chinese HCC cohort. A total of 13 genes were identified as MRD monitoring genes. Among these 13 genes, *TP53*, *TERT*, *CTNNB1*, *APC*, and *CDKN2A* were well-defined driver genes for HCC (41–43, 47). The remaining eight genes have also been experimentally verified to be associated with HCC. *RBM10*, a member of the RNA binding motif gene family, plays a regulatory role in alternative splicing. As a tumor suppressor, its downregulation has been linked to tumor progression, metastasis, and poor prognosis in multiple human cancers, including HCC (48–50). *NTRK3*, located on chromosome 15q25, has been wildly reported as a tumor suppressor implicated in the modulation of cell growth, invasion, and migration in a diverse array of tumors, including HCC (51–53). *NOTCH1* and *NOTCH2* are the key regulators of stem cell proliferation, differentiation, and apoptosis. Numerous studies have revealed that *NOTCH1* and *NOTCH2* are involved in the development of HCC, and their activation contributes to HCC cell growth and aggressiveness and poor overall survival of HCC patients (54–57). *NF1*, one of the largest human genes, is located on chromosome 17, band q11.2.6. Recently, Lu et al. (58) identified *NF1* as a critical driver for lenvatinib resistance in HCC, whose loss reactivates the PI3K/AKT and MAPK/ERK signaling pathways. *CREBBP*, encoding an acetyltransferase, is one of the most frequently mutated genes in small cell lung cancer. In the last few years, some studies documented that CREBBP activation is associated with the early recurrence of HCC, and targeting CREBBP attenuates HCC progression (59, 60). *GLI3*, a member of the Hedgehog signaling pathway, is upregulated in a variety of tumor types (61). The positive correlation of Gli3 with tumor progression has been observed in HCC (62), pancreatic cancer (63), colon cancer (64), ovarian cancer (65), breast cancer (65), and bladder cancer (66). *EZH2* is the catalytic subunit of polycomb repressive complex 2, which methylates histone H3 lysine 27, silencing a number of tumor-suppressor genes, including E-cadherin. Available data have supported EZH2 involved in metastatic spread and tumor angiogenesis. Several recent studies not only linked this role of EZH2 to HCC development but also bridged its pro-oncogenic function to the decrease of PD-L1 and shaping of tumor immunosuppressive microenvironment, suggesting that HCC patients with *EZH2* mutations should be treated with immunotherapy carefully (67–69). MRD positivity was defined as ≥ one MRD monitoring gene mutation in this fixed panel.

We further evaluated the predictive value of MRD status for post-surgery relapse in an independent HCC cohort and showed that patients who were MRD-positive postoperatively had a significantly high risk of early relapse versus those with negative MRD. The prognostic value of MRD positivity has been well-established as an independent prognostic factor for poor RFS. MRD status’s sensitivity and specificity to predict early postoperative relapse were 75% and 100%, respectively. These results showed that this panel provides a cost-effective and feasible approach to monitoring MRD and predicting early postoperative relapse. However, our data also indicated that in some cases, MRD detection at a single postoperative time point was insufficient to predict the prognosis of HCC patients, which might result from inadequate ctDNA release from MRD right after curative resection. Serial MRD monitoring could overcome this limitation and provide a full picture of MRD dynamics during the course of HCC.

Our study has several limitations. First, this is a retrospective study, and the screening strategy for MRD monitoring genes is developed by ourselves, which is reasonable but may not be strict enough. Nevertheless, our results are still beneficial to promoting a standardized sequencing process and a rigorous strategy for identifying MRD monitoring genes. Second, the cohort for MRD performance evaluation is relatively small, and serial ctDNA sampling is in a few patients. Further prospective studies focusing on large serial samplings will be needed to evaluate the performance of this fixed panel in detail.

## Conclusion

In summary, this study demonstrates the feasibility of MRD evaluation based on a tumor-agnostic liquid biopsy approach for HCC patients and reports promising data. The established 13-gene panel can reliably predict early relapse after radical hepatectomy, contributing to the personalized management of HCC patients.

## Data availability statement

The original contributions presented in the study are included in the article/**Supplementary Material**. Further inquiries can be directed to the corresponding author/s.

## Ethics statement

The study was approved by the ethical institutional review board of Zhujiang Hospital and performed following the guidelines of the Declaration of Helsinki. Written informed consent was obtained from individual participants.

## Author contributions

Conception and design: MP, YC, YX, TC, and KZ. Provision of study materials or patients: MP. Collection and assembly of data: YX, TC, KZ, YW, LC, GH, HL, CZ, and SF. Data analysis and interpretation: YX, JiaPC, KZ, TC, JinPC, XZ, CC, MH, and KZ. Drafting of the manuscript: JinPC, TC, YX, JiaPC, and KZ. Review and/or revision of the manuscript: All authors. Accountable for all aspects of the work: All authors. All authors contributed to the article and approved the submitted version.
