# Supplementary material for: Plasma-only circulating tumor DNA analysis detects minimal residual disease and predicts early relapse in hepatocellular carcinoma patients undergoing curative resection
Source: Front Oncol. 2023 Mar 7;13:1119744. doi: 10.3389/fonc.2023.1119744 (PMC10028131; doi:10.3389/fonc.2023.1119744)
Supplement: Supplementary file 1 [file DataSheet_1.pdf]

Supplementary Material

Supplementary Fig. 1 The flowchart of the study

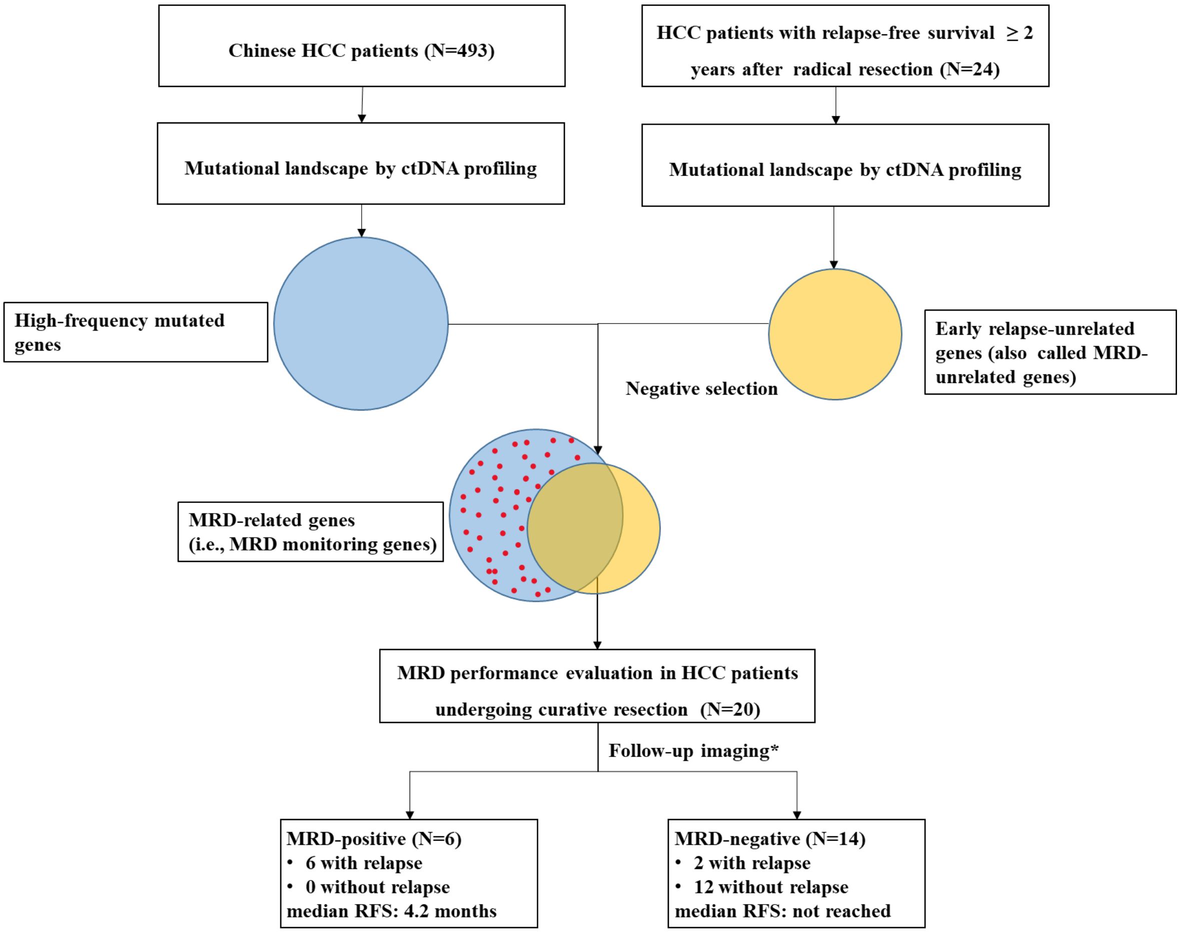

Abbreviations: HCC: hepatocellular carcinoma; ctDNA: circulating tumor DNA; MRD: minimal residual disease; RFS: relapse-free survival.

*Patients underwent postoperative follow-up imaging following the Chinese Society of Clinical Oncology (CSCO) guideline, regardless of MRD status. According to the CSCO guideline, imaging was performed every 3-6 months for 2 years, then every 6 months.

Supplementary Fig. 2 Venn diagram representing the number of genes shared by the 381-gene and 733-gene NGS panels

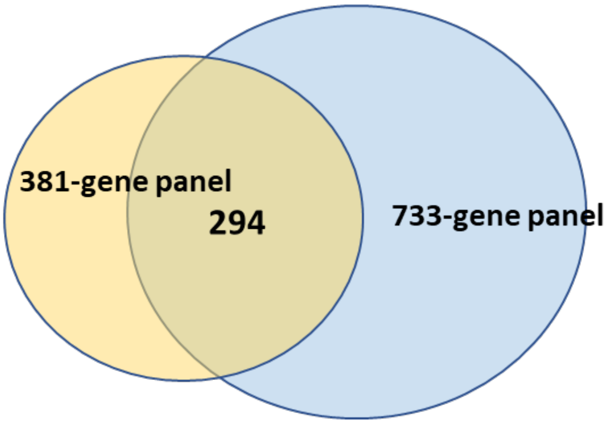

Supplementary Table 1. Gene list of the 381-gene NGS panel

ABL1, ABL2, ACVR1B, ACVR2A, ADAM29, ADGRA2, AKT1, AKT2, AKT3, ALK, AMER1, APC, AR, ARAF, ARFRP1, ARID1A, ARID1B, ARID2, ASXL1, ATM, ATR, ATRX, AURKA, AURKB, AXIN1, AXL, BAP1, BARD1, BCL2, BCL2L1, BCL2L11, BCL2L2, BCL6, BCOR, BCORL1, BCR, BIRC5, BLK, BLM, BMX, BRAF, BRCA1, BRCA2, BRD4, BRIP1, BTG1, BTK, CARD11, CBFB, CBL, CCND1, CCND2, CCND3, CCNE1, CD274, CD79A, CD79B, CDC73, CDH1, CDK12, CDK4, CDK6, CDK8, CDKN1A, CDKN1B, CDKN2A, CDKN2B, CDKN2C, CEBPA, CHD2, CHD4, CHEK1, CHEK2, CIC, CRBN, CREBBP, CRKL, CRLF2, CSF1R, CSK, CSNK1A1, CTCF, CTNNA1, CTNNB1, CUL3, CXCR4, CYLD, CYP2C19, CYP2D6, DAXX, DDR1, DDR2, DICER1, DNMT3A, DOT1L, DPYD, EGF, EGFR, EMSY, EP300, EPHA2, EPHA3, EPHA5, EPHA7, EPHB1, ERBB2, ERBB3, ERBB4, ERCC1, ERG, ERRFI1, ESR1, ETV1, ETV4, ETV5, ETV6, EZH2, FAM135B, FAM46C, FANCA, FANCC, FANCD2, FANCE, FANCF, FANCG, FANCL, FAS, FAT1, FBXW7, FGF10, FGF14, FGF19, FGF23, FGF3, FGF4, FGF6, FGFR1, FGFR2, FGFR3, FGFR4, FGR, FH, FLCN, FLT1, FLT3, FLT4, FOXL2, FOXP1, FRS2, FUBP1, FYN, GABRA6, GATA1, GATA2, GATA3, GATA4, GATA6, GID4, GLI1, GLI2, GLI3, GNA11, GNA13, GNAQ, GNAS, GRIN2A, GRM3, GSK3B, H3F3A, HCK, HGF, HNF1A, HRAS, HSD3B1, HSP90AA1, IDH1, IDH2, IGF1R, IGF2, IKBKE, IKZF1, IL7R, INHBA, INPP4B, IRF2, IRF4, IRS2, ITK, JAK1, JAK2, JAK3, JUN, KAT6A, KDM5A, KDM5C, KDM6A, KDR, KEAP1, KEL, KIT, KLHL6, KMT2A, KMT2C, KMT2D, KRAS, LCK, LIMK1, LMO1, LRP1, LRP1B, LYN, LZTR1, MAGI2, MAP2K1, MAP2K2, MAP2K4, MAP3K1, MAP4K5, MCL1, MDM2, MDM4, MED12, MEF2B, MEN1, MET, MITF, MLH1, MPL, MRE11, MS4A1, MSH2, MSH6, MST1R, MTOR, MUTYH, MYB, MYC, MYCL, MYCN, MYD88, NEK11, NF1, NF2, NFE2L2, NFKBIA, NKX2-1, NOTCH1, NOTCH2, NOTCH3, NPM1, NRAS, NRG1, NRG3, NSD1, NTRK1, NTRK2, NTRK3, NUP93, PAK3, PALB2, PARK2, PAX5, PBRM1, PDCD1LG2, PDGFRA, PDGFRB, PDK1, PIK3C2B, PIK3CA, PIK3CB, PIK3CD, PIK3CG, PIK3R1, PIK3R2, PKD2, PLA2G1B, PLCG2, PMS2, POLD1, POLE, PPP2R1A, PRDM1, PREX2, PRKAR1A, PRKCI, PRKDC, PRSS8, PTCH1, PTEN, PTK2, PTK6, PTPN11, QKI, RAC1, RAD50, RAD51, RAF1, RANBP2, RARA, RB1, RBM10, RET, RICTOR, RIT1, RNF43, ROCK1, ROCK2, ROS1, RPTOR, RUNX1, RUNX1T1, RXRA, SDHA, SDHB, SDHC, SDHD, SETD2, SF3B1, SIK1, SLIT2, SMAD2, SMAD3, SMAD4, SMARCA2, SMARCA4, SMARCB1, SMO, SNCAIP, SOCS1, SOX10, SOX2, SOX9, SPEN, SPOP, SPTA1, SRC, SRMS, STAG2, STAT3, STAT4, STK11, STK24, SUFU, SYK, TAF1, TBX3, TCF7L2, TEK, TERT, TET2, TGFBR1, TGFBR2, TIE1, TMPRSS2, TNFAIP3, TNFRSF14, TNFSF11, TNK2, TOP1, TOP2A, TP53, TPMT, TSC1, TSC2, TSHR, TYK2, U2AF1, UGT1A1, VEGFA, VHL, WEE1, WEE2, WISP3, WT1, XIAP, XPO1, YES1, ZBTB2, ZNF217, ZNF703, ZNF750

Supplementary Table 2. Gene list of the 733-gene NGS panel

ABCB11, ABI1, ABL1, ABRAXAS1, ACKR3, ACSL3, ACVR1, ACVR1B, ACVR2A, AEN, AFF3, AFF4, AKT1, AKT2, AKT3, ALK, ALKBH2, ALKBH3, AMER1, ANK1, APC, APEX1, APEX2, APLF, APOBEC3B, APTX, AR, ARAF, AREG, ARHGAP5, ARID1A, ARID1B, ARID2, ARNT, ASXL1, ATM, ATP1A1, ATP2B3, ATR, ATRIP, ATRX, AURKA, AXIN1, AXIN2, AXL, B2M, BAP1, BARD1, BAZ1A, BCL10, BCL11A, BCL11B, BCL2, BCL2L1, BCL6, BCOR, BCORL1, BIRC3, BIRC5, BLM, BMP5, BMPR1A, BRAF, BRCA1, BRCA2, BRD4, BRIP1, BTG1, BTK, BUB1B, CACNA1D, CALR, CAMTA1, CANT1, CARD11, CARS, CASP8, CBFA2T3, CBFB, CBL, CBLB, CCDC6, CCNB1IP1, CCND1, CCND2, CCND3, CCNE1, CCNH, CCNO, CD274, CD79A, CD79B, CDC73, CDH1, CDH10, CDH11, CDK12, CDK2, CDK4, CDK6, CDK7, CDK8, CDKN1A, CDKN1B, CDKN1C, CDKN2A, CDKN2B, CDKN2C, CDX2, CEBPA, CENPS, CENPX, CETN2, CHAF1A, CHD1, CHD2, CHD4, CHEK1, CHEK2, CHIC2, CIC, CIITA, CLIP1, CLK2, CLTCL1, CNBP, CNOT3, COL7A1, CRBN, CREB3L1, CREB3L2, CREBBP, CRKL, CRLF2, CRNKL1, CRTC1, CRTC3, CSF1R, CSF3R, CTCF, CTNNB1, CTNND2, CTR9, CUL1, CUL3, CUL4A, CUL5, CUX1, CXCR4, CYLD, CYP17A1, CYSLTR2, DAXX, DCLRE1A, DCLRE1B, DCLRE1C, DDB1, DDB2, DDIT3, DDR2, DDX10, DDX3X, DDX5, DDX6, DICER1, DIS3, DIS3L2, DKC1, DMC1, DNM2, DNMT1, DNMT3A, DNTT, DOCK8, DROSHA, DUT, EBF1, EED, EGFR, EIF3E, EIF4A2, ELANE, ELF3, ELF4, ELK4, ELL, ELOA, EME1, EME2, EMSY, ENDOV, EP300, EPAS1, EPCAM, EPHA2, EPHA3, EPHA7, EPHB1, EPS15, ERBB2, ERBB3, ERBB4, ERC1, ERCC1, ERCC2, ERCC3, ERCC4, ERCC5, ERCC6, ERCC8, EREG, ERF, ERRFI1, ESR1, ETNK1, ETV6, EWSR1, EXO1, EXT1, EXT2, EZH2, EZR, FAAP100, FAAP20, FAAP24, FAH, FAM135B, FAM47C, FAN1, FANCA, FANCB, FANCC, FANCD2, FANCE, FANCF, FANCG, FANCI, FANCL, FANCM, FAS, FAT1, FAT4, FBXW7, FEN1, FES, FGF19, FGF3, FGF4, FGFR1, FGFR2, FGFR3, FGFR4, FH, FHIT, FLCN, FLT1, FLT3, FLT4, FOXA1, FOXL2, FOXP1, FRK, FRS2, FUBP1, FUS, G6PD, GALNT12, GAS7, GATA1, GATA2, GATA3, GBA, GEN1, GFI1, GJB2, GLI1, GLI2, GLI3, GNA11, GNA13, GNAQ, GNAS, GPC3, GRB2, GREM1, GRIN2A, GSK3B, GSTT1, GTF2H1, GTF2H3, GTF2H4, GTF2H5, H2AFX, H3F3A, HDAC1, HDAC2, HELQ, HES1, HEY1, HFE, HFM1, HGF, HIF1A, HIP1, HIST1H3B, HLA-A, HLA-B, HLA-C, HLTF, HMBS, HMGA2, HMGB1, HNF1A, HNRNPA2B1, HOOK3, HOXA11, HOXB13, HRAS, HUS1, HUS1B, IDH1, IDH2, IGF1R, IGF2, IKBKE, IKZF1, IL6ST, IL7R, INPP4B, IRS2, ITGAV, ITK, JAK1, JAK2, JAK3, JMJD1C, JUN, KCNJ5, KDM5A, KDM5C, KDM6A, KDR, KEAP1, KIT, KLF4, KMT2A, KMT2C, KMT2D, KNL1, KRAS, LASP1, LATS1, LATS2, LCK, LEF1, LIFR, LIG1, LIG3, LIG4, LMNA, LMO1, LRP1B, LZTR1, MAD2L2, MAP2K1, MAP2K2, MAP2K4, MAP3K1, MAPK1, MAX, MBD4, MCL1, MDC1, MDM2, MDM4, MECOM, MED12, MEF2B, MEN1, MET, MGA, MGMT,

MITF, MLH1, MLH3, MLLT3, MLST8, MMS19, MNAT1, MPG, MPL, MPLKIP, MRE11, MSH2, MSH3, MSH4, MSH5, MSH6, MTAP, MTOR, MUS81, MUTYH, MYB, MYC, MYCL, MYCN, MYD88, MYOD1, NAB2, NABP2, NBN, NCOA3, NCOR1, NCOR2, NDRG1, NEIL1, NEIL2, NEIL3, NF1, NF2, NFE2L2, NFIB, NFKBIA, NHEJ1, NHP2, NKX2-1, NME1, NONO, NOP10, NOTCH1, NOTCH2, NOTCH3, NOTCH4, NPM1, NRAS, NRG1, NRG3, NSD2, NSD3, NT5C2, NTHL1, NTRK1, NTRK2, NTRK3, NUDT1, NUP93, OGG1, PAK1, PALB2, PARP1, PARP2, PARP3, PARP4, PAX3, PAX5, PAX7, PAX8, PBRM1, PCDH9, PCNA, PDCD1LG2, PDGFB, PDGFRA, PDGFRB, PDPK1, PER1, PER2, PER3, PHF6, PHOX2B, PICALM, PIK3CA, PIK3CB, PIK3CD, PIK3R1, PIK3R2, PIK3R3, PIM1, PLCG2, PLXNA1, PLXNB1, PML, PMS1, PMS2, PNKP, POLB, POLD1, POLD3, POLD4, POLE, POLE2, POLE3, POLE4, POLG, POLH, POLI, POLK, POLL, POLM, POLN, POLQ, POT1, POU2AF1, POU5F1, PPARG, PPM1D, PPP2R1A, PPP2R2A, PPP4R1, PPP4R2, PPP4R3A, PPP4R3B, PPP4R4, PPP6C, PRCC, PRDM1, PRDM16, PRDM9, PREX2, PRF1, PRKACA, PRKAR1A, PRKCH, PRKDC, PRPF19, PRSS1, PSIP1, PTCH1, PTEN, PTK2, PTK6, PTPN11, PTPN13, PTPRD, PTPRT, QKI, RAC1, RAD1, RAD18, RAD21, RAD23A, RAD23B, RAD50, RAD51, RAD51B, RAD51C, RAD51D, RAD52, RAD54B, RAD54L, RAD54L2, RAD9A, RAD9B, RAF1, RANBP2, RAP1GDS1, RARA, RASA1, RB1, RBBP8, RBM10, RBX1, RDM1, RECQL, RECQL4, RECQL5, RET, REV1, REV3L, RFC1, RFC2, RFC3, RFC4, RFC5, RFWD3, RGS7, RHBDF2, RHEB, RHOA, RHOH, RICTOR, RIF1, RIT1, RMI1, RMI2, RNF168, RNF213, RNF4, RNF43, RNF8, ROS1, RPA1, RPA2, RPA3, RPA4, RPS6KA3, RPS6KB1, RPTOR, RRM2B, RUNX1, RUNX1T1, RXRA, SBDS, SDC4, SDHA, SDHAF2, SDHB, SDHC, SDHD, SEM1, SERPINA1, SERPINB3, SETBP1, SETD2, SETMAR, SF3B1, SFPQ, SGK1, SH2B3, SH2D1A, SHOC2, SHPRH, SLC25A13, SLC29A1, SLC34A2, SLC45A3, SLIT2, SLX1A, SLX4, SMAD2, SMAD3, SMAD4, SMARCA1, SMARCA2, SMARCA4, SMARCB1, SMO, SMUG1, SOCS1, SOS1, SOX2, SOX9, SPEN, SPO11, SPOP, SPRED1, SPRTN, SPTA1, SRC, SRGAP3, SRSF2, SRY, STAG2, STAT3, STK11, SUFU, SUZ12, SYK, TBL1XR1, TBX3, TCF3, TCF7L2, TCL1A, TDG, TDP1, TDP2, TEAD2, TELO2, TERT, TET1, TET2, TFE3, TGFBR1, TGFBR2, THBS2, TIMELESS, TMEM127, TMEM189, TMPRSS2, TNFAIP3, TOP2A, TOP3A, TOP3B, TOPBP1, TP53, TP53BP1, TP63, TRAF7, TREX1, TREX2, TRIM37, TSC1, TSC2, TSHR, TSPAN31, TYK2, U2AF1, UBE2A, UBE2B, UBE2N, UBE2T, UBE2V2, UNG, UROD, USP1, USP6, USP8, UVSSA, VEGFA, VHL, WAS, WDR48, WIF1, WRN, WT1, XAB2, XPA, XPC, XPO1, XRCC1, XRCC2, XRCC3, XRCC4, XRCC5, XRCC6, YAP1, ZBTB16, ZFHX3, ZNF217, ZNF479, ZNF703, ZNF750, ZNRF3

Supplementary Table 3. Basic characteristics of HCC patients in the ZJ2020 cohort.

**Clinicopathologic factors**	**No. of Patients (%)**
**Gender**	
Female	4 (16.7%)
Male	20 (83.3%)
**Age**	
<50	11 (45.8%)
≥50	13 (54.2%)
**HBV**	
Negative	5 (20.8%)
Positive	19 (79.2%)
**HCV**	
Negative	23 (95.8%)
Positive	1 (4.2%)
**Largest tumor diameter**	
<5cm	17 (70.8%)
≥5cm	7 (29.2%)
**Tumor number**	
Multiple	5 (20.8%)
Single	19 (79.2%)
**Liver cirrhosis**	
No	15 (62.5%)
Yes	9 (37.5%)
**AFP**	
≤400 ng/ml	17 (70.8%)
>400 ng/ml	7 (29.2%)
**MVI**	
NA	1 (4.2%)
No	18 (75.0%)
Yes	5 (20.8%)
**PVTT**	
No	24 (100.0%)
**BCLC**	
A	13 (54.2%)
B	6 (25.0%)
C	5 (20.8%)
**Child pugh**	
A	23 (95.8%)
B	1 (4.2%)
**CNLC**	
I	15 (62.5%)
II	4 (16.7%)<br>5 (20.8%)
III	

Abbreviations: HBV: hepatitis B virus; HCV: hepatitis C virus; AFP; alpha-foetoprotein; MVI: microvascular invasion; PVTT: portal vein tumor thrombosis; BCLC: Barcelona Clinic Liver Cancer Staging; CNLC: China Liver Cancer Staging; NA: not available.
